# Genome-wide identification of the walnut MYC gene family and functional characterization of Xinjiang wild walnut under low-temperature stress

**DOI:** 10.3389/fgene.2024.1399721

**Published:** 2024-05-13

**Authors:** Ya-Ting Song, Kai Ma, Yu Zhao, Li-Qun Han, Li-Qiang Liu

**Affiliations:** ^1^ College of Horticulture, Xinjiang Agricultural University, Urumqi, China; ^2^ Xinjiang Key Laboratory of Genome Research and Genetic Improvement of Specialty Fruits and Vegetables, Xinjiang Institute of Horticultural Crops, Xinjiang Academy of Agricultural Sciences, Xinjiang Regional Scientific Observatory and Experiment Station of Fruit Trees, Ministry of Agriculture, Urumqi, China

**Keywords:** walnut, MYC transcription factor, bioinformatics, subcellular localization, low-temperature stress

## Abstract

**Introduction:** MYC transcription factors are the basic regulators of the jasmonic acid signaling pathway and play important roles in plant growth and development and the response to adverse stress. In recent years, severe winter freezing and late spring frost in the main planting area of walnut in Xinjiang have affected the growth and development of walnut, which has become a prominent problem restricting walnut production. Xinjiang wild walnut is the only remaining wild species of walnuts in China, which contains a lot of genes with excellent traits, and is important for the cultivation and breeding.

**Methods:** In this paper, the physicochemical properties and bioinformatics of MYC transcription factor members in walnut were analyzed, and the nine MYC were screened from the transcriptome data under low temperature stress. At last, we study the subcellular localizations and the expression patterns of the nine MYC members in Xinjiang wild walnut.

**Results:** The results revealed that 30 MYC members were identified from published walnut whole-genome data, and their evolutionary relationships with Arabidopsis and poplar were divided into six groups according to clustering analysis, among which *JrMYC22 and JrMYC23* had high homology with *PtrMYC2b*, which is induced by jasmonic acid in response to low-temperature stress. Walnut MYC members are unevenly distributed on 12 chromosomes. The prediction of promoter cis-acting elements of walnut MYC transcription factor family members revealed that cis-acting elements related to jasmonic acid and lowtemperature stress were the ones with the greatest number of members, with 12. In addition, all nine MYC family members in Xinjiang wild walnut plants responding to low-temperature stress exhibited strong fluorescence responses in the nucleus. The expression levels of these members in response to low-temperature stress revealed that *JrMYC28, JrMYC31, JrMYC33, JrMYC34, and JrMYC35* were highly expressed, and it was hypothesized that *JrMYC28, JrMYC31, JrMYC33, JrMYC34, and JrMYC35* might play a key role in the response to lowtemperature stress.

**Discussion:** The results of this study provide a theoretical basis for further research on the functional mechanisms of the MYC transcription factor family members in walnut.

## 1 Introduction

The jasmonate signaling pathway is relatively complex and involved in plant growth and development, biosynthesis, phytohormone signaling and the regulation of resistance (Creelman and Mullet, 1995; León and Sánchez-Serrano, 1999); this pathway regulates downstream functional genes in response to adverse stress through the specific binding of transcription factors to related *cis*-acting elements to improve plant resistance (Ali and Baek, 2020). COI1 (CORONATINE INSENSITIVE1) and JAZ (Jasmonate-ZIM) are coreceptors of the jasmonate signaling pathway, in which JA-Ile binds directly to JAZ and COI1, and JAZ protein ubiquitination is degraded by the 26S proteasome and at the same time activates related transcription factors, which induce the expression of downstream functional genes in response to adverse stresses, such as low temperature, drought (PEG), and high salinity (Peñuelas et al., 2019; Ruan et al., 2019), in plants. MYC transcription factors play a key role in the jasmonic acid signaling pathway and can specifically bind to multiple target genes in response to adversity (Wang et al., 2023).

MYC transcription factors, a subfamily of the bHLH family, are core regulators of the jasmonate signaling pathway. MYC transcription factors have a conserved bHLH structural domain at the C-terminal end and a bHLH_MYC_N structural domain at the N-terminal end, which has a DNA-binding function that allows the bHLH structural domains to form a homo or heterodimeric composite substance (Boter et al., 2004; Kazan and Manners, 2013). Among them, MYC2 is the most studied member of the MYC transcription factor family, which can specifically bind to JAZ proteins and is involved in the regulation of jasmonic acid signaling, response to pathogens, and resistance to adverse stresses (Yanfang et al., 2018). In *Arabidopsis thaliana*, MYC2 has a DNA binding specificity similar to that of MYC3 and MYC4, and they interact to activate the jasmonic acid signaling pathway and regulate plant responses to adverse stress (Fernández-Calvo et al., 2011; Schweizer et al., 2013; Gao et al., 2016; Wang et al., 2017; Wang et al., 2021). Overexpression of AtMYC2, AtMYC67, and AtMYC70 activates the promoters of downstream functional genes to increase plant resistance in response to dehydration, high-salt, and low-temperature stresses (Abe et al., 1997; Shinozaki and Yamaguchi-Shinozaki, 2007) in apple, and overexpression of MdMYC2 in response to low-temperature stress increases the expression of *MdCIbHLH1, MdCBF1, MdCBF2*, and *MdCBF3* to enhance plant cold resistance (Wang et al., 2019). In tomato, MYC2 induces the expression of the ethylene gene *SlERF. B8*, which triggers the jasmonic acid signaling pathway, plays a regulatory role in increasing the cold tolerance of tomato plants (Ding et al., 2022). In seedlings of Bupleurum chinensis, BcMYC2 was regulated through the jasmonic acid signaling pathway in response to drought stress, which affected the biosynthesis mechanism of Bupleurum chinensis (Wang et al., 2021). In Arabidopsis, overexpression of AtMYC2 was able to increase plant resistance to Botrytis cinerea (Fu et al., 2020). MYC transcription factors play important regulatory roles in plant growth and development and the response to adverse stresses, and one of them plays a key role in the response to low-temperature stress. Currently, the MYC transcription factor family has been identified in plants such as wheat (Bai et al., 2019), tomato (Feng et al., 2023), poplar (Hu et al., 2023), and tea tree (Chen et al., 2021). However, bioinformatic analysis of the MYC transcription factor family in walnut has not been reported.

Walnut (*Juglans regia* L.) belongs to the walnut family of deciduous walnut trees, is one of the world’s four major nut species, is also an important species of woody oilseed, and has high medicinal, health and nutritional value. Many places in our country are planted and widely distributed geographically. However, in recent years, the main planting area of Xinjiang walnut has suffered winter frost damage, the frequency of spring late frost damage has increased, the loss of water and drying of walnut light branches, flower buds, new fruits, young fruits and other tissues have suffered damage, and severe tree or even whole plant death has seriously affected the growth and development of walnut plants; thus, walnut plants have become a constraint on the production of outstanding problems (McCully et al., 2004; Han et al., 2022). Therefore, it is necessary to study the cold tolerance mechanism of walnut plants under low-temperature stress and to screen and identify genes that respond to low-temperature stress. Xinjiang wild walnut is the only remaining wild species of walnut in China and is an endangered protected plant in China; it contains many genes for excellent traits and is an important gene pool for the study of walnut cultivation and breeding (Zhang et al., 2020; Li et al., 2023). In this study, 30 walnut MYC family members were screened from published walnut genome-wide data, and bioinformatic analyses of their physicochemical properties, phylogenetic relationships, conserved motifs, chromosomal structural localization, promoter *cis*-acting elements, and protein interactions were performed. Wild walnut seedlings from Xinjiang were subjected to low-temperature (4°C) stress treatment, and the expression of nine MYC family members in response to low-temperature stress was analyzed via transcriptome data. MYC family members, and the subcellular localization of MYC family members in Xinjiang wild walnut was determined by tobacco injection. At the same time, real-time fluorescence was used to quantitatively analyze the expression patterns of MYC members in different tissues, such as roots, stems and leaves, under low-temperature stress, with the aim of laying a theoretical foundation for in-depth research on the functional mechanisms of walnut MYC transcription factor family members.

## 2 Materials and methods

### 2.1 Materials and treatments

The test material was wild walnut seedlings (JF) from Xinjiang, which were planted at the Institute of Horticultural Crops, Xinjiang Academy of Agricultural Sciences, and transplanted into organic matter-rich soil and peat soil (2:1) when the seedlings grew well. When the seedlings grew to five compound leaves, the well-grown plants with consistent growth were screened and placed in an artificial climate chamber (4°C, 16 h light) for low-temperature stress treatment. Three to five leaves under the terminal leaf were taken at 0 h (control) and 8 h of treatment, with three biological replicates for each treatment, and then the leaves were placed in an ultralow-temperature refrigerator at −80°C after quick freezing in liquid nitrogen to carry out transcriptome sequencing via the Illumina sequencing platform. Transcriptome sequencing using the Illumina sequencing platform.

At the same time, well-grown plants with consistent growth were screened and subjected to 4°C low-temperature stress treatment, and 2-5 functional leaves under the parietal leaves and tissue materials of roots, stems, and leaves were removed at 0 h, 1 h, 2 h, 4 h, 8 h, 12 h, and 24 h of treatment, respectively, with three biological replicates for each treatment. The plants were subsequently cleaned and placed in liquid nitrogen quickly frozen and then put into −80°C ultralow-temperature freezers for storage.

### 2.2 Identification of the walnut MYC transcription factor family

The whole-genome sequence of walnut (PRJNA291087) was downloaded from NCBI (https://www.ncbi.nlm.nih.gov/bioproject/291087). Hidden Markov models of MYC proteins (PF14215 and PF00010) were downloaded from the Pfam (http://pfam.xfam.org) database, and all MYC protein sequences in the annotated protein files of walnut were compared using TBtools V2.069 software. After that, the protein sequence of each MYC gene in walnut was determined based on the protein database accession number, and to ensure the accuracy of the conserved structural domains of the screened walnut MYC genes, Candidate protein sequences were subjected to structure prediction using the online software NCBI-CDD (https://www.ncbi.nlm.nih.gov/cdd/) and SMART (http://smart.emblheidelberg.de/), removing sequences that did not contain the bHLH and bHLH_ MYC_N structural domains, non-full-length and repetitive sequences to identify the MYC transcription factors of walnut.

### 2.3 Physicochemical properties of the walnut MYC transcription factor family

In order to determine the MYC transcription factor family members in walnuts, refer to Hu(Hu et al., 2023). The basic physicochemical properties of walnut MYC proteins were analysed using the online tool ProtParam (https://web.expasy.org/protparam/), including amino acid length (aa), molecular mass (Da), isoelectric point (pI), hydrophilicity and instability index and protein hydrophobicity. Hydrophobicity, etc.; subcellular localisation of walnut MYC transcription factors was analysed using the online software WoLF PSORT (https://wolfpsort.hgc.jp/).

### 2.4 Construction of a phylogenetic tree of the walnut MYC transcription factor family

Amino acid sequences of eight members of the MYC gene family were obtained from the *Arabidopsis thaliana* (https://www.arabidopsis.org/) database; amino acid sequences from the Mauve poplar genome database V4.1 were downloaded using the online website Phytozome (https://phytozome-next.jgi.doe.gov/) sequences, downloaded the Hidden Markov Models of MYC proteins (PF14215 and PF00010) using the Pfam (http://pfam.xfam.org) database, and obtained the amino acid sequences of the poplar MYC transcription factor family by comparing the sequences of all MYC proteins in the poplar annotated protein files using TBtools V2.069; the software Clustal W was used to compare the walnut with Arabidopsis and poplar MYC transcription factors of walnut and *Arabidopsis thaliana* and poplar using the software Clustal W. Multiple sequence comparison was performed; the protein sequences of the MYC transcription factor families of the three species were calculated using MEGA 7.0 software, and the phylogenetic tree was constructed using the neighbor-joining (NJ) method, with the following execution parameters: BootStrap method 1000; Poisson model; pairwise deletion.

### 2.5 Analysis of conserved structural domains of the walnut MYC transcription factor family

The conserved structural domains of MYC proteins from walnut were analyzed using the online software MEME (http://meme-suite.org/tools/meme), with the number of motif searches set to 10 and the others set to default parameters.

### 2.6 Chromosomal position analysis and gene duplication analysis of the MYC transcription factor family in walnut

The positional information of the MYC transcription factor family members was extracted from the annotation files of the walnuts, and the positions of the walnut MYC transcription factor family members on the chromosomes were mapped using the online software MG2C_v2.1 (http://mg2c.iask.in/mg2c_v2.1/). Genome-wide replication events were obtained using the One Step MCScanX tool in TBtools software (Chen et al., 2020), and the gene duplication relationships of JrMYCs were determined.

### 2.7 Analysis of the promoters and cis-acting elements of the walnut MYC transcription factor family

The promoter sequences 2000 bp upstream of the walnut MYC transcription factor family members were obtained using TBtools software, and the promoter *cis*-acting elements were later utilized in the online software Plantcare (http://bioinformatics.psb.ugent.be/webtools/plantcare/html) for predictions and visualization of the output using TBtools software.

### 2.8 Walnut MYC transcription factor family protein interaction network

The prediction of receptor-interacting proteins was performed using the STRING database (https://string-db.org/) on the protein sequences of the MYC transcription factor family members of walnut, with the species parameter selecting the model plant *Arabidopsis thaliana* and a threshold value of 0.4.

### 2.9 Construction and subcellular localization analysis of MYC transcription factor family members in Xinjiang wild walnut

Sequences of each member of the MYC transcription factor family of Xinjiang wild walnut were amplified, stop codons were removed, and the sequences were ligated into a transient expression pCAM35-GFP vector with a GFP fluorescent signal. The primers used were designed with the online tool Vazyme (https://crm.vazyme.com/cetool/simple.html). The constructed target gene plasmid was subsequently transformed into *Agrobacterium tumefaciens* GV3101 via the conventional freeze‒thaw method. Afterward, the Agrobacterium sap containing the target gene (OD value 0.8–1) was centrifuged at 5,000 rpm for 10 min, the supernatant was removed, the injection buffer for transient expression of tobacco was added, and the mixture was allowed to stand at room temperature for 2 h. Four-week-old tobacco plants were injected from the lower epidermis of the leaves by using a 1 mL syringe, the position was marked, the leaves were incubated in the dark for 24 h, and the fluorescence of the leaves was directly observed by using a laser confocal microscope.

### 2.10 RNA extraction and real-time fluorescence quantitative analysis

Different tissue materials, such as the roots, stems and leaves of Xinjiang wild walnut plants subjected to low-temperature (4°C) stress, were collected, and the total RNA of the test materials was extracted using a Plant Total RNA Extraction Kit (Tiangen, Beijing, China). The quality and purity of the RNA were detected by 1% agarose gel electrophoresis and UV spectrophotometry. Afterward, 1 µg of each sample was reverse transcribed by using a High-Capacity cDNA Reverse Transcription Kit (Thermo Fisher Scientific, Shanghai, China) to reverse transcribe the RNA from a 1 µg sample, which was subsequently stored at −20°C. Specific primers were designed using the online tool Vazyme (https://crm.vazyme.com/cetool/simple.html) to screen the transcriptome data for nine MYC transcription factor family members in response to low-temperature stress (Supplementary Table S1), and ACT 7 was used as an internal reference to design specific primers for the nine MYC transcription factors using a qTOWER3 G fluorescence quantitative PCR instrument (Analytikjena, Germany) and SYBR Green qPCR Master Mix kit (Xavier, Wuhan) methods to analyze the gene expression levels of seedlings in different tissues and in response to low-temperature stress. The qPCR mixture consisted of 7.5 µL of qPCR mix, 0.75 µL of upstream and downstream primers, 2 µL of cDNA template, and 15 µL of ddH_2_O water. The following reaction program was used: predenaturation at 95°C for 10 min, denaturation at 95°C for 15 s, annealing at 60°C for 30 s, 40 cycles, melting curve (95–65°C, 0.3°C/15 s), 3 biological replicates, and relative expression was calculated using the 2^−ΔΔCT^ method.

### 2.11 Data analysis

Data statistics and calculations were performed using Office 2022, data variability analysis was performed using SPSS 26 software, and relative expression heatmaps were plotted using Origin 2018 and TBtools software.

## 3 Results

### 3.1 Identification of the walnut MYC transcription factor family

The screening of walnut genome-wide data based on MYC transcription factors with a conserved bHLH structural domain at the C-terminus and a bHLH_MYC_N structural domain at the N-terminus, excluding proteins with deletions or non-full-length fragments, yielded 30 JrMYC genes, named JrMYC1 to JrMYC30, with a greater number of members than in the model plants *Arabidopsis thaliana* (8 AtMYC genes) and poplar (10 AtMYC genes) (Hu et al., 2023).

In this study, 30 members of the MYC transcription factor family were screened in walnut for physicochemical characterization and subcellular localization prediction, and the length of the protein sequences ranged from 484 to 926 aa, with an average of 667.23 aa; the molecular weight of each member ranged from 53.83 to 101.23 kDa, with an average of 74.09 kDa; and the isoelectric point (PI) ranged from 4.82 to 8.09. The isoelectric points (PIs) ranged from 4.82 to 8.09, with an average of 5.67. Among them, there was 1 protein with a PI > 7, accounting for 3.3%, and 29 proteins with a PI < 7, accounting for 96.7%. The instability coefficients ranged from 40.4 to 68.2, with an average of 50.14. The instability coefficients of each member were greater than 40, indicating that walnut MYC proteins may be less stable *in vitro*. The fat coefficients ranged from 67.36 to 87.21, with an average of 78.96. The fat coefficients determine the thermal stability of the protein, indicating that the walnut MYC protein has greater thermal stability. The hydrophilicity predictions were all negative, indicating that the walnut MYC proteins were all hydrophobic. Subcellular localization prediction revealed that the MYC proteins had roles in the nucleus, chloroplast, and endoplasmic reticulum, 27 of which were localized to the nucleus at 90%, 2 of which were localized to the chloroplast at 6.6%, and 1 of which was localized to the endoplasmic reticulum at 3.3% (Table 1).

**TABLE 1:** Basic information on the walnut MYC transcription factor family.

Name	Gene ID	Amino acids (aa)	Molecular Weight (KDa)	Isoelectric points	Instability index	Aliphatic index	Gravy	Location
JrMYC1	XP_018807981	656	73.78	5	55.22	83.55	−0.467	Nucleus
JrMYC2	XP_035549874	637	71.15	5.23	58.43	87.21	−0.365	Nucleus
JrMYC3	XP_018814174	738	81.98	5.24	57.77	77.07	−0.572	Nucleus
JrMYC4	XP_018852488	632	71.22	5.22	56.79	82.18	−0.415	Nucleus
JrMYC5	XP_035549141	714	79.75	5.64	67.39	74.96	−0.642	Nucleus
JrMYC6	XP_018827229	759	83.93	5.83	45.17	81.78	−0.388	Nucleus
JrMYC7	XP_018825262	689	76.42	5.81	47.5	78.46	−0.424	Nucleus
JrMYC8	XP_018825260	757	84.16	5.77	47.8	80.3	−0.39	Nucleus
JrMYC9	XP_018827230	697	76.85	5.84	44.37	80.52	−0.408	Nucleus
JrMYC10	XP_035547567	733	80.93	5.71	43.65	80.95	−0.384	Nucleus
JrMYC11	XP_018827228	760	84.05	5.83	45.45	81.67	−0.392	Nucleus
JrMYC12	XP_018817026	926	101.23	6.16	40.4	81.45	−0.356	Nucleus
JrMYC13	XP_018846265	732	81.52	6.13	41.76	79.13	−0.384	Endoplasmic Reticulum
JrMYC14	XP_035549142	658	73.45	5.53	68.2	75.11	−0.686	Nucleus
JrMYC15	XP_035545463	724	80.75	5.23	50.18	80.14	−0.314	Chloroplast
JrMYC16	XP_018858098	842	92.42	4.82	42.29	78.97	−0.338	Nucleus
JrMYC17	XP_018858097	843	92.61	4.85	42.25	78.41	−0.344	Nucleus
JrMYC18	XP_018824283	845	93.71	4.93	51.06	82.73	−0.316	Nucleus
JrMYC19	XP_018815047	494	55.39	6.01	50.4	82.29	−0.536	Nucleus
JrMYC20	XP_018841495	616	69.3	5.3	50.91	70.26	−0.681	Nucleus
JrMYC21	XP_018841494	659	74.31	5.38	49.78	71.44	−0.583	Chloroplast
JrMYC22	XP_018817566	684	74.84	5.56	53.51	70.29	−0.599	Nucleus
JrMYC23	XP_018811463	652	71.47	5.3	52.91	67.36	−0.653	Nucleus
JrMYC24	XP_018824308	489	54.23	6.05	45.7	85.71	−0.391	Nucleus
JrMYC25	XP_018824309	498	55.67	5.88	52.16	80.62	−0.452	Nucleus
JrMYC26	XP_018860395	494	55.7	5.59	45.19	85.81	−0.434	Nucleus
JrMYC27	XP_018814191	487	54.49	8.09	52.85	76.12	−0.468	Nucleus
JrMYC28	XP_018818377	611	67.83	6.44	48.77	76.78	−0.536	Nucleus
JrMYC29	XP_035548572	484	53.83	6	50.58	80.79	−0.502	Nucleus
JrMYC30	XP_018827272	507	56.02	5.76	45.79	76.88	−0.453	Nucleus

### 3.2 Phylogenetic tree analysis

**FIGURE 1:**
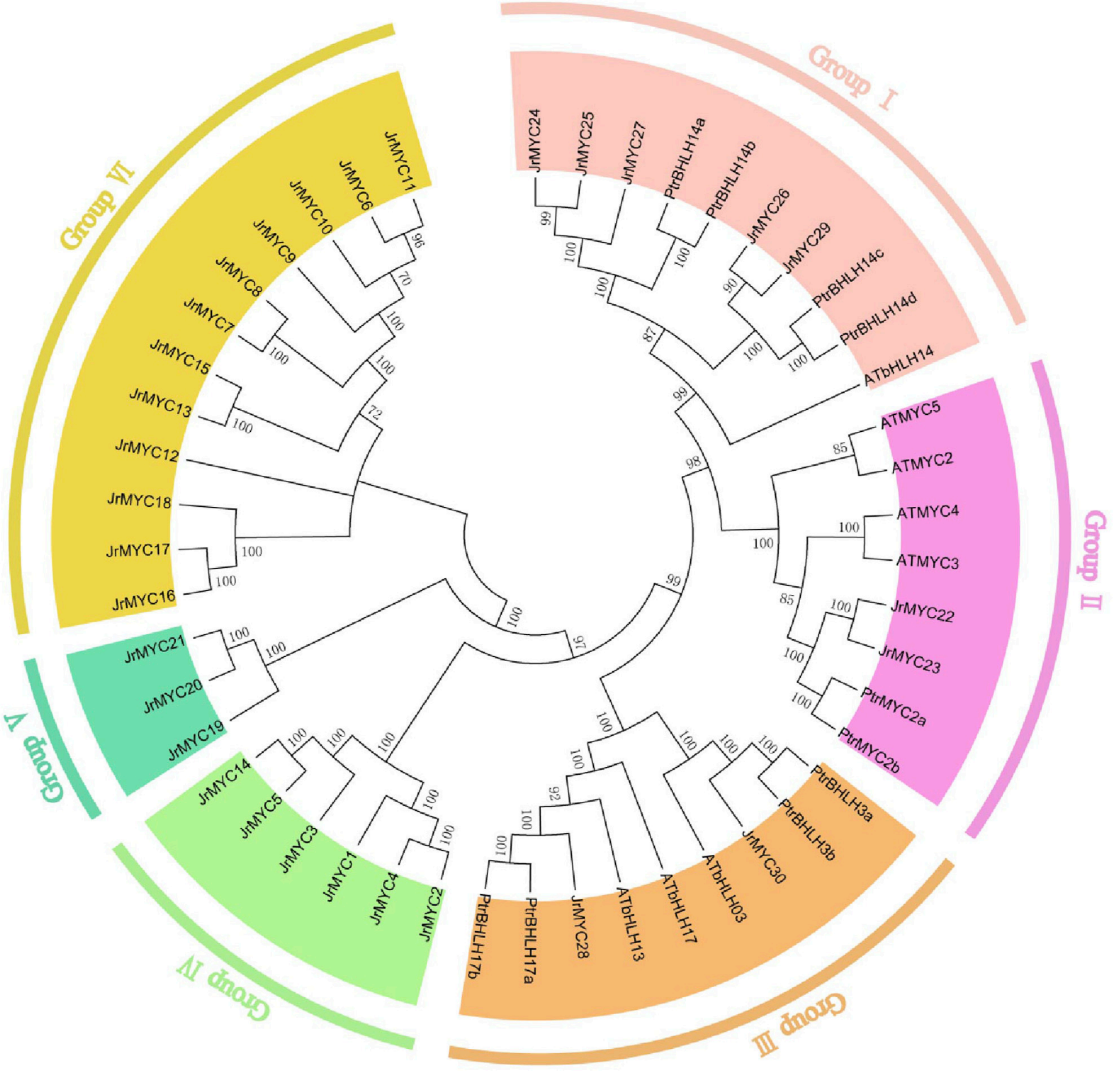
Phylogenetic analysis of the walnut MYC transcription factor family. Different colors represent different groups.

To reveal the evolutionary relationship between the MYC transcription factor families of walnut and other species, evolutionary trees of walnut, *Arabidopsis thaliana* and poplar were constructed based on multiple sequence comparisons using the neighbor–joining (NJ) method in MEGA 7.0 software (Figure 1). The evolutionary tree showed that each member of the MYC transcription factor family of the three species was classified into six subfamilies (Groups I, II, III, IV, V, and VI), and members of the walnut MYC transcription factor family were distributed in all the groups, which contained 5, 2, 2, 6, 3 and 12 walnut MYC proteins, respectively. The branching of members of the walnut MYC transcription factor family varied, and compared to *Arabidopsis thaliana*, walnut is closer in homology to poplar. It has been reported that homology and phylogenetic relationships between species can help to predict the potential functions of genes (Zhang et al., 2020). Currently, in the study of the poplar MYC transcription factor family, we found that the expression of the *PtrMYC14a and PtbHLH14b* genes could respond to high salt and drought stresses (Hu et al., 2023), and that of *JrMYC24*, *JrMYC25, and JrMYC27* was close to that of *PtrMYC14a and PtbHLH14b*; therefore, we hypothesized that *JrMYC24*, *JrMYC25*, and *JrMYC27* could respond to high salt and drought stresses. Can respond to high salt and drought stresses. *PtrMYC2b* gene expression is induced by jasmonic acid and responds to low temperature and high salt stresses (Hu et al., 2023). The evolutionary tree shows that *JrMYC22* and *JrMYC23* have high homology with *PtrMYC2b*, and it is hypothesized that *JrMYC22* and *JrMYC23* may have both cold resistance and salt resistance.

### 3.3 Chromosome localization analysis

**FIGURE 2:**
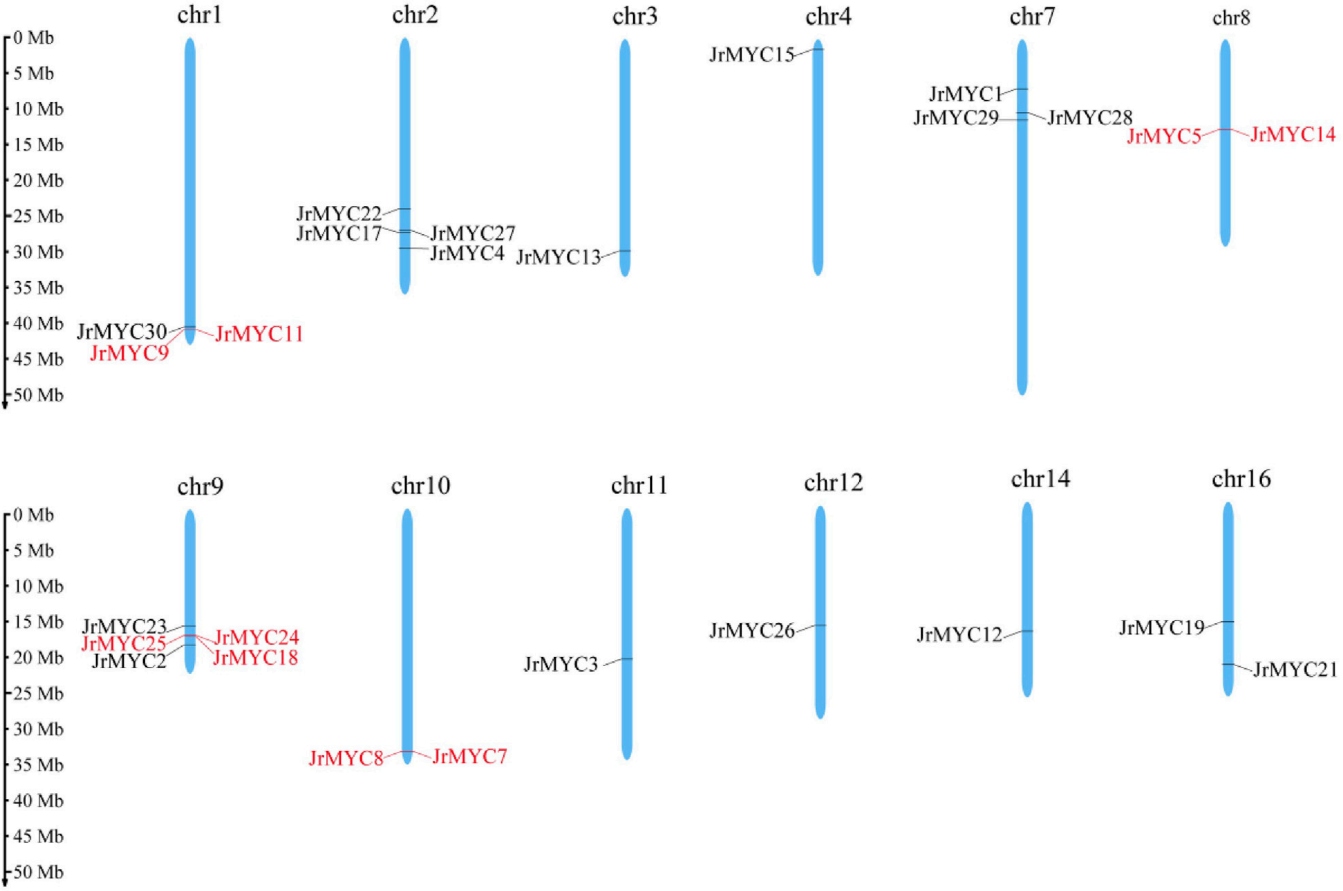
Chromosomal distribution of the walnut MYC gene family. Red indicates genes in which tandem duplication occurred.

Based on the whole walnut genome and annotation information, the chromosomal localization of MYC in the walnut variety was mapped using the MG2C online website. The map shows that various members of the walnut MYC transcription factor family are located on chromosomes Chr1, Chr2, Chr3, Chr4, Chr7, Chr8, Chr9, Chr10, Chr11, Chr12, Chr14, and Chr16, with Chr1 (*JrMYC9, JrMYC11, JrMYC30*), Chr2 (*JrMYC4, JrMYC17, JrMYC22, JrMYC27*), Chr7 (*JrMYC1, JrMYC28, JrMYC29*), Chr8 (*JrMYC5, JrMYC14*), Chr9 (*JrMYC2, JrMYC18, JrMYC23, JrMYC24, JrMYC25*), Chr10 (*JrMYC7, JrMYC8*), and Chr16 (*JrMYC19, JrMYC21*) having two or more JrMYC genes (Figure 2). Genes on chromosomes with distances less than 100 kb are tandemly duplicated (Kapli et al., 2020). Analysis revealed tandem duplication of the walnut MYC transcription factor members *JrMYC9 and JrMYC11* on Chr1; *JrMYC5 and JrMYC14* on Chr8; *JrMYC18, JrMYC24, and JrMYC25* on Chr9; and *JrMYC7 and JrMYC8* on Chr10.

**FIGURE 3:**
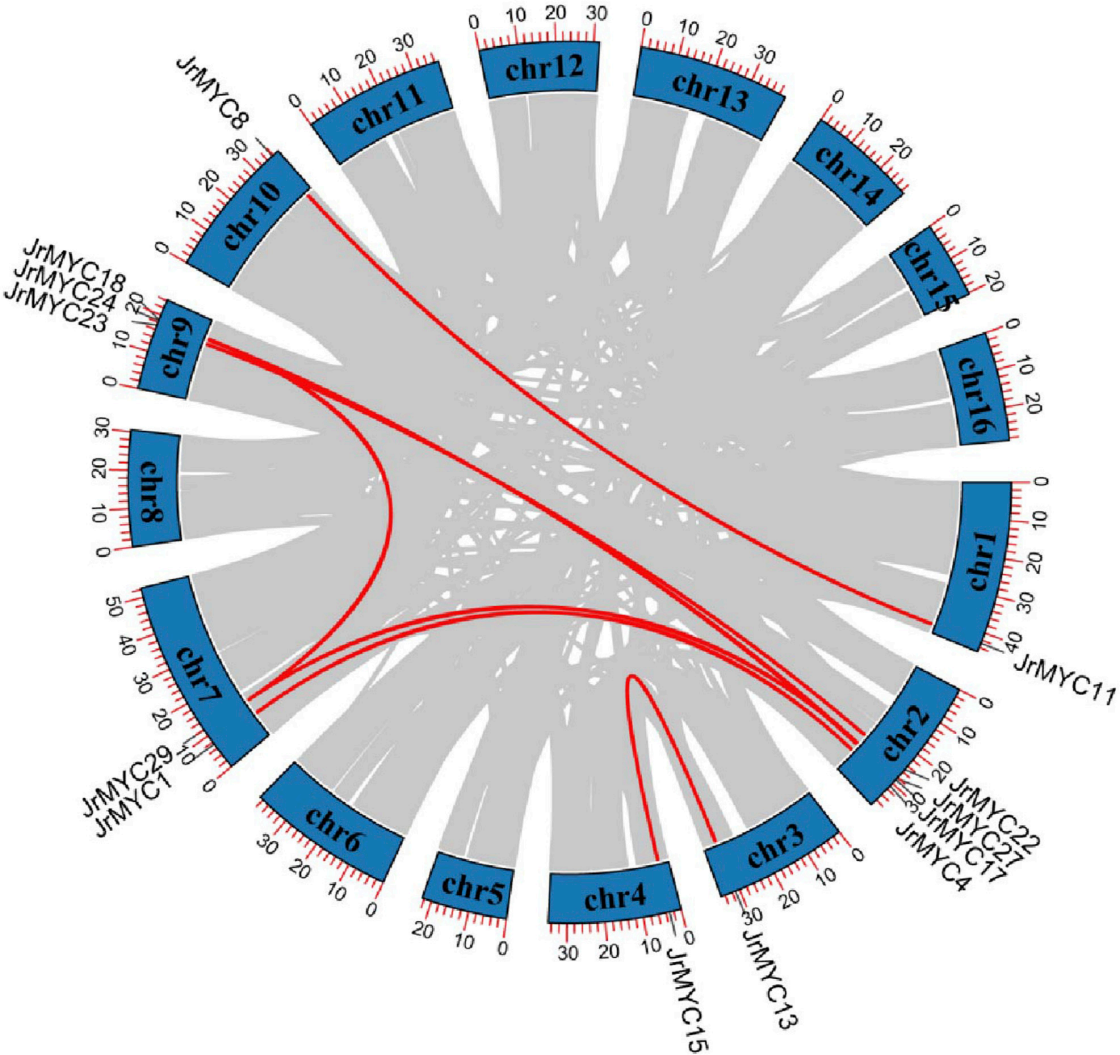
Gene replication analysis of MYC transcription factor family members in walnut. The chromosome number is shown on each chromosome, and the red line represents 8 pairs of paralogous genes: *JrMYC8* and *JrMYC11*, *JrMYC1* and *JrMYC4*, *JrMYC27* and *JrMYC29*, *JrMYC22* and *JrMYC23*, *JrMYC24* and *JrMYC27*, *JrMYC17* and *JrMYC18*, *JrMYC13* and *JrMYC15*, *JrMYC24* and *JrMYC29*.

Gene replication is the main method of gene recombination and amplification and can potentially affect the evolution and adaptation of species. In this study, collinearity analysis of the walnut MYC transcription factor family was conducted by using TBtools software (Figure 3), and 13 members of the walnut MYC transcription factor family exhibited gene repetition events, indicating that the walnut MYC transcription factor family has diverse functions.

### 3.4 Phylogenetic developmental tree and sequence structure analysis of walnut MYC proteins

**FIGURE 4:**
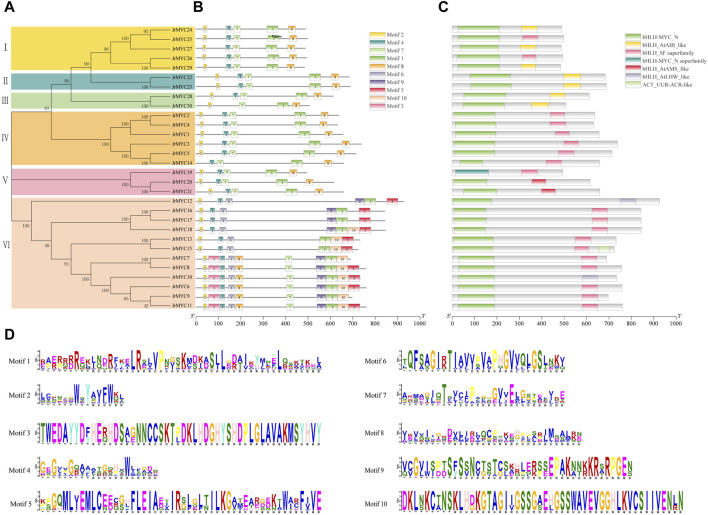
Phylogenetic developmental tree of walnut MYC proteins, motif composition, conserved structural domains and motif sequence signatures. **(A)** A phylogenetic developmental tree of the walnut MYC transcription factor family was constructed using the NJ method. **(B)** Motif identification of walnut MYC transcription factor family members using the MEME online website, with numbers 1–10 representing different motifs. **(C)** Conserved structural domains of walnut MYC transcription factor family members. **(D)** Sequence signatures of the 10 motifs, with the horizontal coordinate indicating the amino acid with the highest frequency of occurrence and the vertical coordinate indicating the frequency corresponding to the amino acid of occurrence.

To further analyze the motif composition and conserved structural domains of walnut MYC transcription factor family members. First, the protein sequences of 30 members were phylogenetically analyzed using MEGA 7.0 software (Figure 4A), and the members were classified into six subfamilies (Groups I, II, III, IV, V, and VI), in which the genes had a similar topology to the phylogenetic developmental tree of the MYC transcription factor family constructed for three plants (Figure 1). Then, the conserved structural domains of the 30 MYC proteins were predicted using the MEME online website, with the motif value set to 10, and analyzed (Figures 4B–D). All the structural domains of the walnut MYC proteins appeared to be highly conserved during the evolutionary process, and Motif1, Motif2, and Motif4 were distributed among all the members. Among them, Motif1, Motif4, Motif2, Motif3, Motif5, and Motif9 are the conserved characteristic structural domains of the MYC family members bHLH and bHLH_MYC_N, respectively. In addition, conserved structural domains are essential for transcription factor regulation and are able to maintain the tertiary structural conformation of proteins, and the DNA recognition and binding process plays a key role in the binding process of DNA recognition. The conserved structural motifs of most of the walnut MYC genes share a high degree of similarity, and it is hypothesized that there may be functional redundancy in these genes. The JrMYC12, JrMYC13, JrMYC15, JrMYC16, JrMYC17, and JrMYC18 proteins lacked Motif7 and Motif8, and it was hypothesized that these genes might have different functional response mechanisms.

### 3.5 Analysis of promoter cis-acting elements

**FIGURE 5:**
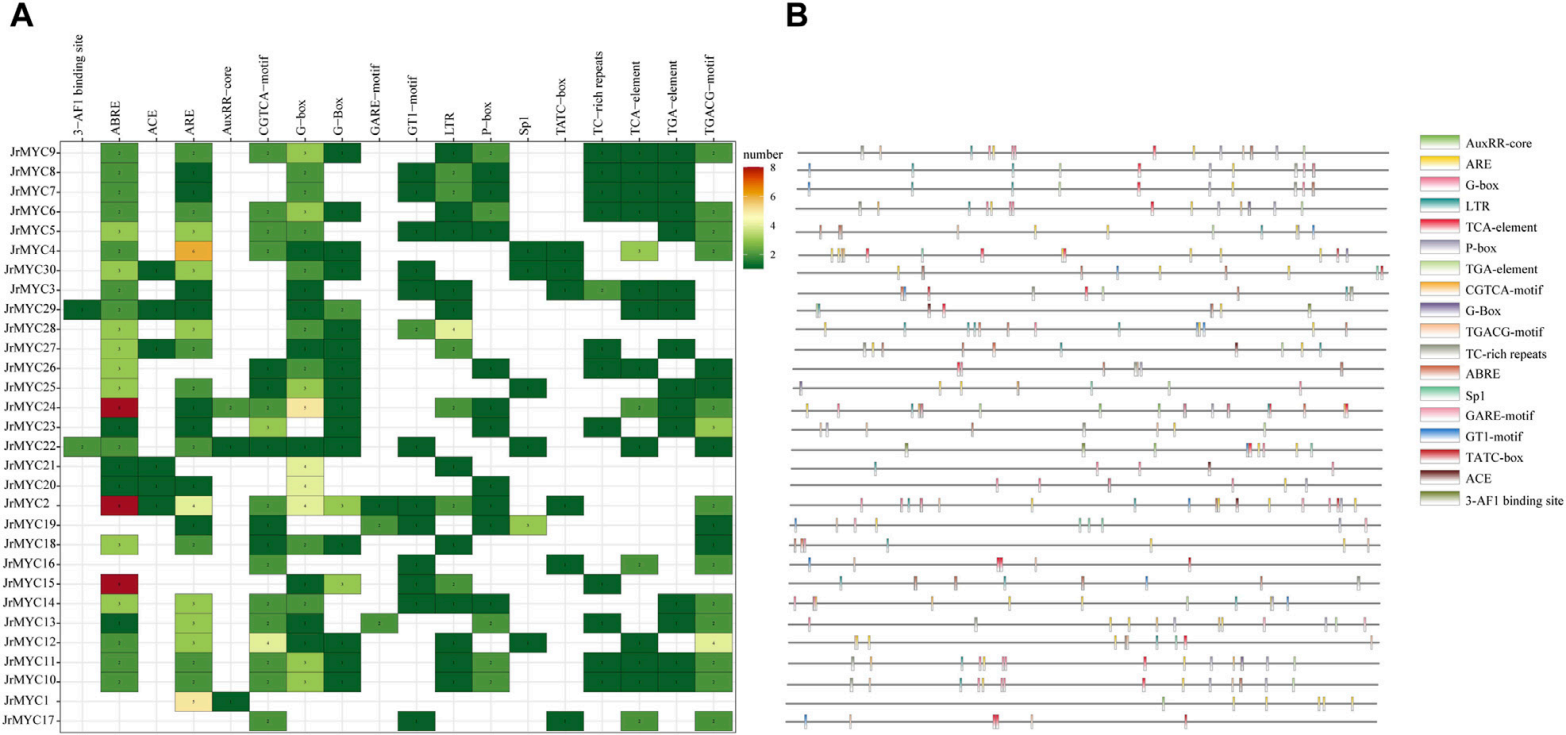
Predicted promoter *cis*-acting elements of walnut MYC transcription factor family members. **(A)** Statistics of the number of each member of the walnut MYC transcription factor family containing promoter *cis*-acting elements. **(B)** The location and type of promoter *cis*-acting elements of each member of the walnut MYC transcription factor family.

Prediction of promoter *cis*-acting elements for each member of the walnut MYC transcription factor family using the PlantCare database revealed that there were 18 genes with promoter *cis*-acting elements related to functions such as light response, hormone response, adversity stress, binding sites, and biosynthesis, and hormone-related *cis*-acting elements were the most numerous and widely distributed. Among them, 15 members contained growth hormone-responsive *cis*-acting elements, 3 with an AuxRR core and 15 with a TGA element; 15 members contained salicylic acid-responsive *cis*-acting elements (TCA elements); 15 members contained gibberellin-responsive *cis*-acting elements; 19 members contained jasmonic acid-responsive *cis*-acting elements; 19 members contained a CGTCA motif and a TGACG motif; 26 members contained abscisic acid response *cis*-acting elements (ABREs); 17 members contained *cis*-acting elements involved in defense and stress responses; 6 members contained TC-rich elements; 12 members had a TATC box; 18 members contained low-temperature response *cis*-acting elements (LTRs); and light-responsive *cis*-acting elements were more abundant, including G-boxes, Sp1s, GT motifs, ACEs and 3-AF1 binding sites, with a total of 26 members containing these elements (Figure 5).

### 3.6 Protein interaction analysis of walnut MYC transcription factor family members

**FIGURE 6:**
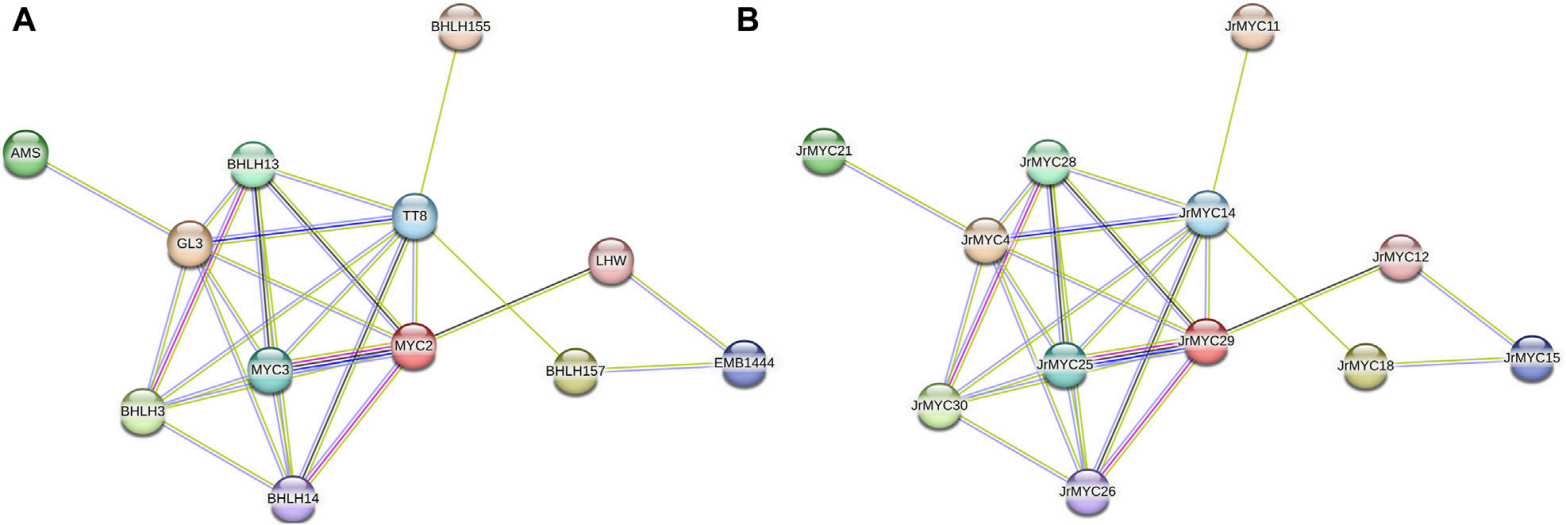
Protein interaction network map of walnut MYC transcription factor family members. **(A)** Predicted protein interactions of walnut MYC transcription factor family proteins with Arabidopsis as the reference species and named after homologous proteins in Arabidopsis. **(B)** Predicted protein interaction map of walnut MYC transcription factor family proteins using *Arabidopsis thaliana* as the reference species and named after walnut.

To further investigate the protein interactions among the members of the walnut MYC transcription factor family, the STRING database (https://string-db.org/) was used to predict the interactions among 30 walnut MYC transcription factor family members after redundancy (Figure 6), with the model plant *Arabidopsis thaliana* serving as the reference species. The results showed that most of the walnut MYC transcription factor family proteins were able to interact with each other, among which 12 walnut MYC transcription factor family members had close protein homology with Arabidopsis, such as MYC2, MYC3, bHLH3, bHLH13, bHLH14, bHLH155, bHLH157, GL3, AMS, TT8, EMB1444, and LHW in Arabidopsis, Based on the number of reciprocal lines, MYC2 and MYC3 were found to have the strongest reciprocal network relationships, and MYC2 and MYC3 were reciprocal homologous proteins with JrMYC25 and JrMYC29 in walnut. It has been shown that in *Arabidopsis thaliana*, MYC2 has similar DNA-binding specificity to MYC3 and MYC4, which interact to activate the jasmonic acid signalling pathway and regulate the plant response to adversity stress (Niu et al., 2011; Schweizer et al., 2013; Gao et al., 2016), and that overexpressed AtMYC2, AtMYC67, and AtMYC70 are induced by dehydration, high salt, and low-temperature stresses to activate the promoters of downstream functional genes in order to enhance plant resistance (Abe et al., 1997; Shinozaki and Yamaguchi-Shinozaki, 2007). Taken together, this suggests that walnut JrMYC25 and JrMYC29 may be hypothesised to function similarly to their Arabidopsis homologues, and that the prediction of protein interactions through members of the walnut MYC transcription factor family could help to further explore the functions played by walnut proteins.

### 3.7 Analysis of the expression pattern of the MYC transcription factor family in walnut under low-temperature stress

**FIGURE 7:**
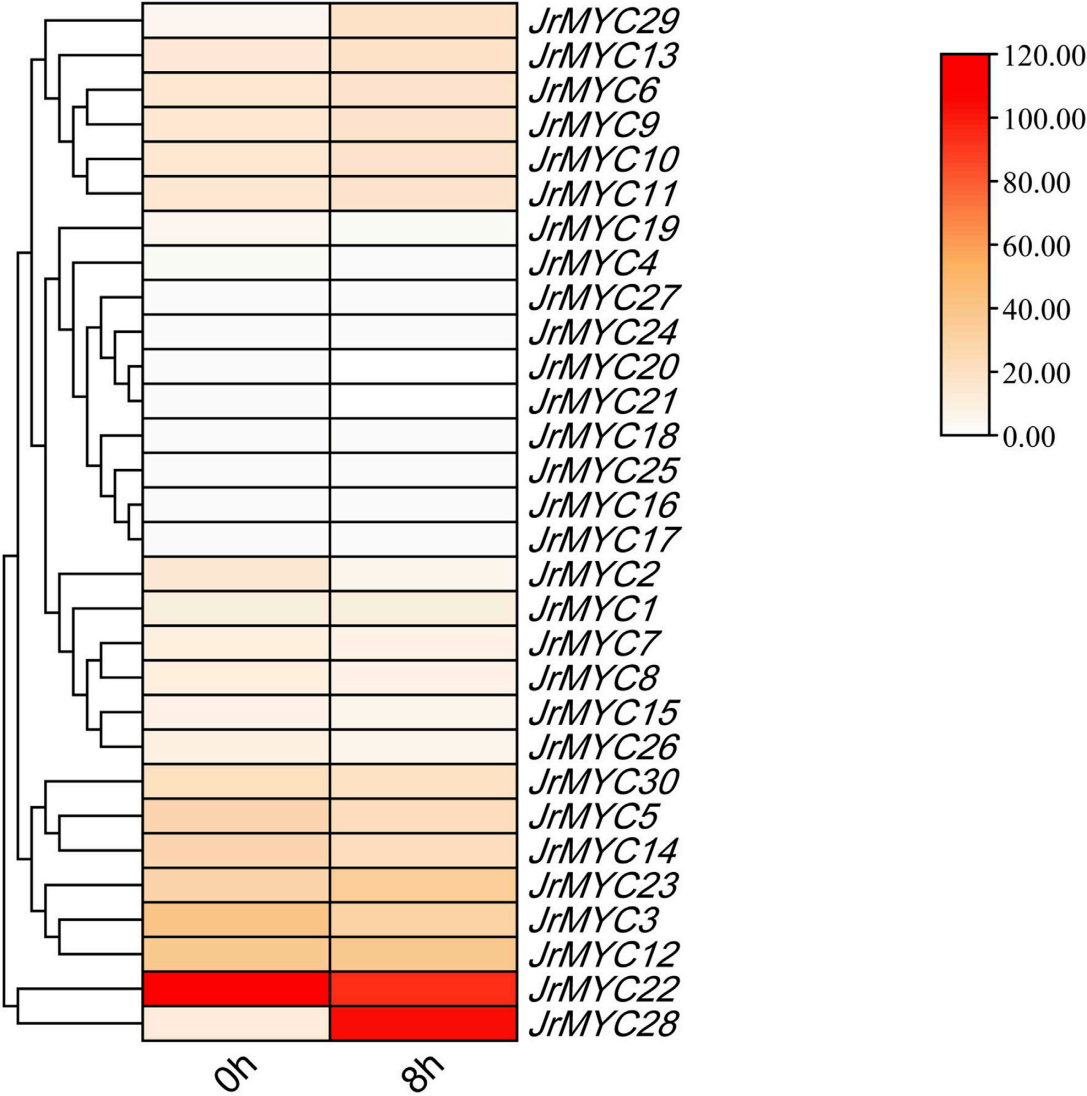
Relative expression of MYC transcription factor family members in the transcriptome.

Transcriptome data analysis of walnut plants under low-temperature stress revealed (Figure 7) that most of the members of the MYC transcription factor family exhibited high levels of expression, and a few members exhibited low levels of expression. Among these genes, *JrMYC22* and *JrMYC28* were highly expressed, *JrMYC22* was highly expressed at 0 h and 8 h, and *JrMYC28* was highly expressed at 8 h. These findings suggest that *JrMYC28* may play a key role in the response to low-temperature stress and that members of the MYC transcription factor family are critical for regulating the response to low-temperature stress.

### 3.8 Subcellular localization of MYC transcription factor family members in Xinjiang wild walnut

**FIGURE 8:**
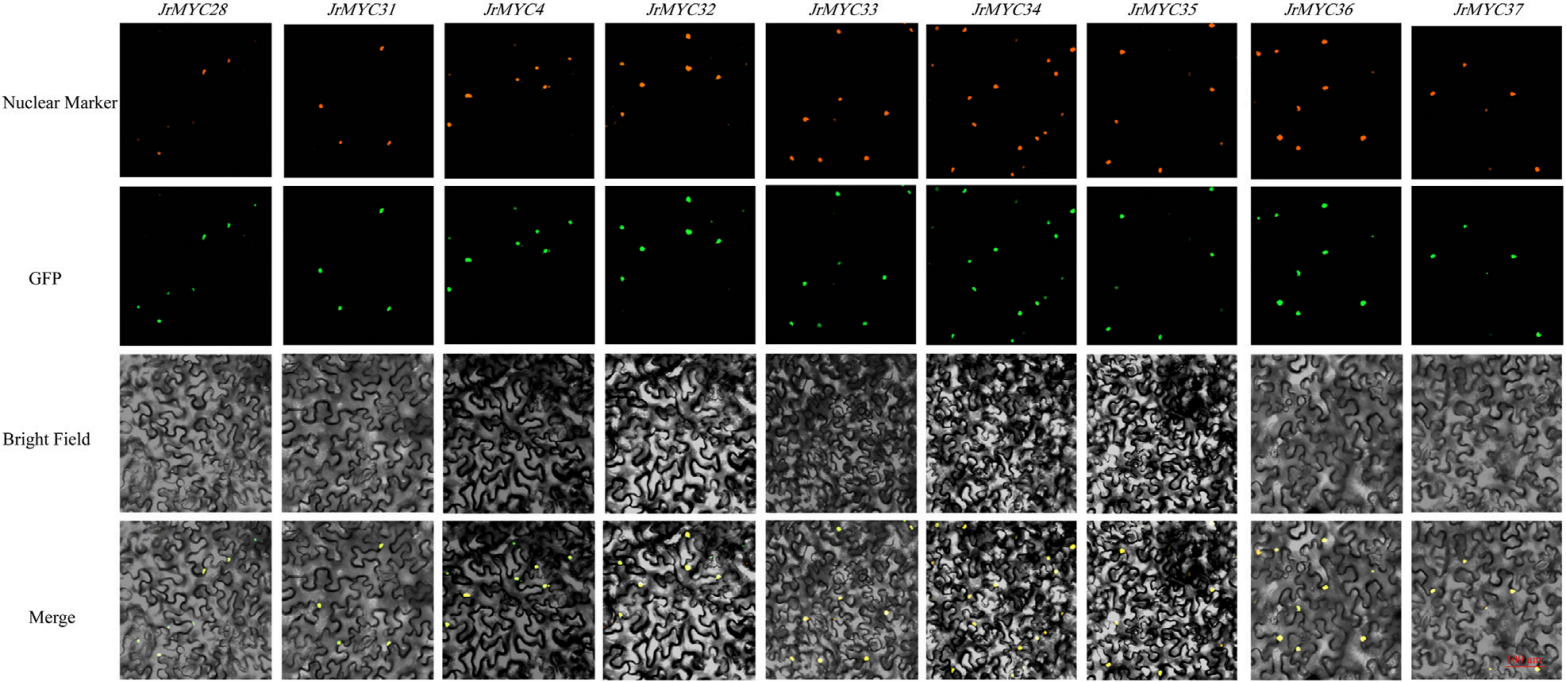
Subcellular localization of MYC transcription factor family members in Xinjiang wild walnut; scale bar, 100 μm.

The transcriptome data of Xinjiang wild walnut plants in response to low-temperature stress were utilized to screen nine MYC transcription factor family members, namely, *JrMYC28, JrMYC31, JrMYC4, JrMYC32, JrMYC33, JrMYC34, JrMYC35, JrMYC36*, and *JrMYC37*. To verify the predicted subcellular localization of the nine MYC transcription factor family members. Therefore, in this study, the gene sequences of these members were cloned and ligated into the pCAM35-GFP vector with GFP fluorescent signals, allowing these genes to be fused to pCAM35-GFP and transiently expressed in tobacco. Subcellular localization revealed that these members were significantly fluorescent in the nucleus, and these nine genes had high expression levels in the nucleus, suggesting that the nucleus may be the site where members of the walnut MYC transcription factor family mainly function (Figure 8).

### 3.9 Expression patterns of MYC transcription factor family members in wild walnuts from Xinjiang under low-temperature stress

**FIGURE 9:**
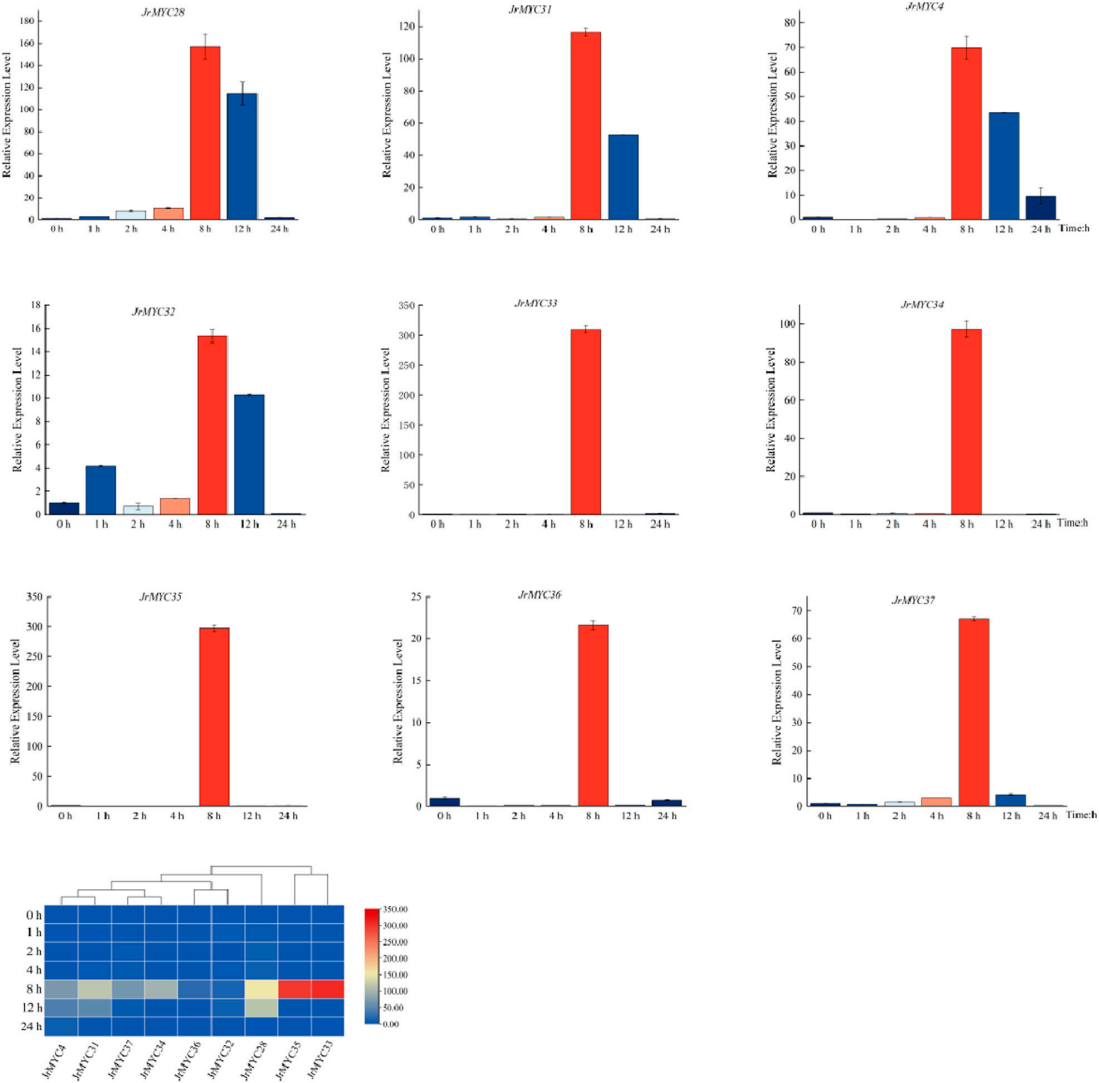
Expression levels of MYC transcription factor family members in wild walnut plants in Xinjiang in response to low-temperature stress.

To further understand the expression patterns of MYC transcription factor family members in Xinjiang wild walnut screened by transcriptome analysis under low-temperature stress, fluorescence quantitative PCR was used to determine the expression levels of the members in response to low-temperature stress at different time intervals in Xinjiang wild walnut seedlings treated at 4°C for 0 h, 1 h, 2 h, 4 h, 8 h, 12 h and 24 h (Figure 9). The results showed that all of these walnut MYC transcription factor family members reached the highest expression level at 8 h after low-temperature stress, and there was variability in the response time and fold increase in the expression of these members. Moreover, the relative expression of these genes in Xinjiang wild walnut increased, among which *JrMYC28, JrMYC31, JrMYC33, JrMYC34 and JrMYC35* in Xinjiang wild walnut exhibited increased expression at 8 h. Taken together, this suggests that *JrMYC28*, *JrMYC31*, *JrMYC33*, *JrMYC34* and *JrMYC35* are hypothesised to potentially play key roles in responding to low-temperature stress.

### 3.10 Expression patterns of MYC transcription factor family members in different tissues of wild walnut plants from Xinjiang

**FIGURE 10:**
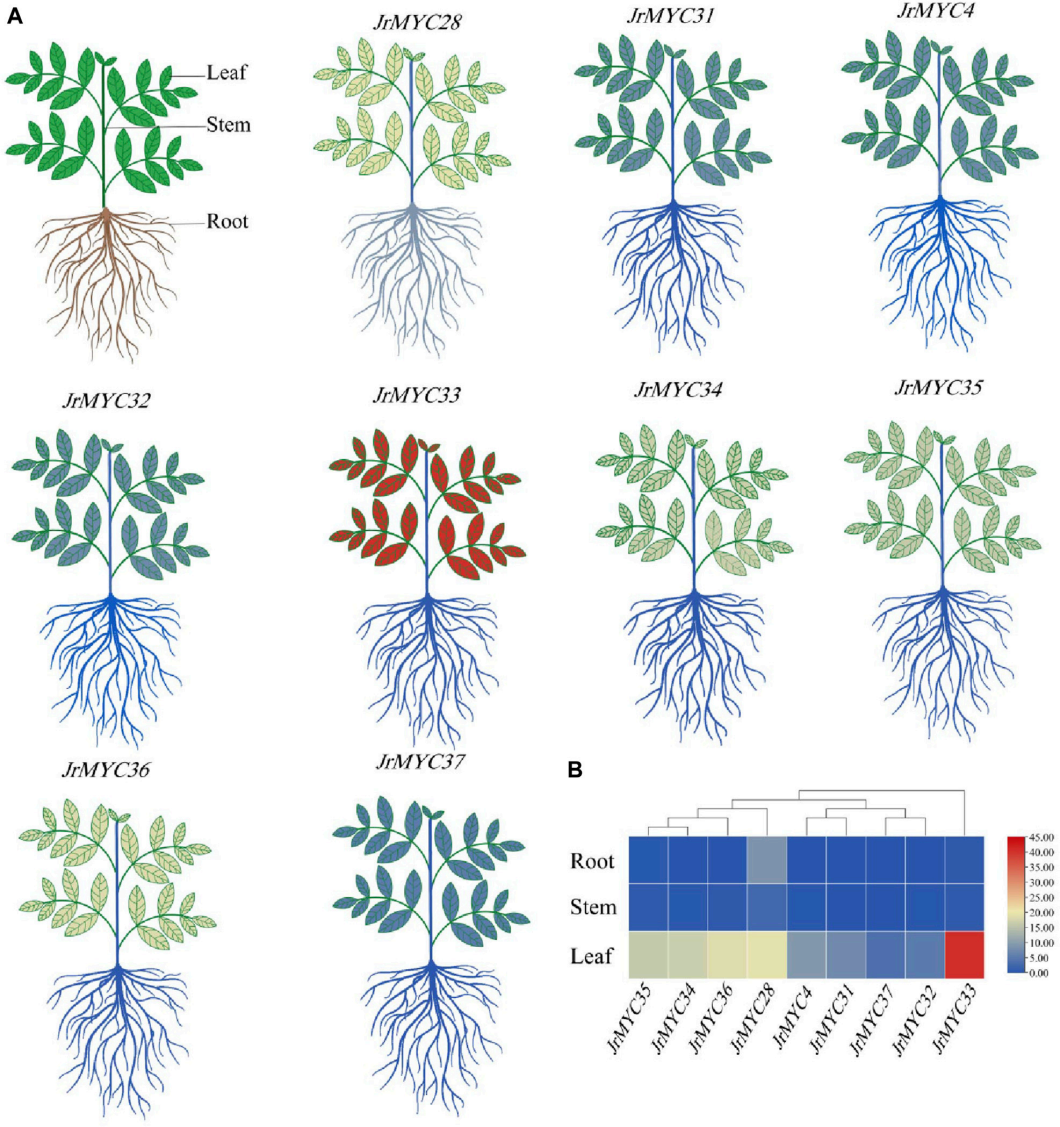
Expression levels of MYC transcription factor family members in different tissues of Xinjiang wild walnut. **(A)** Structural thermograms of different tissues of annual walnut seedlings. **(B)** Heatmaps were plotted as the mean values by TBtools software, with red representing high expression levels and blue representing low expression levels.

**FIGURE 11:**
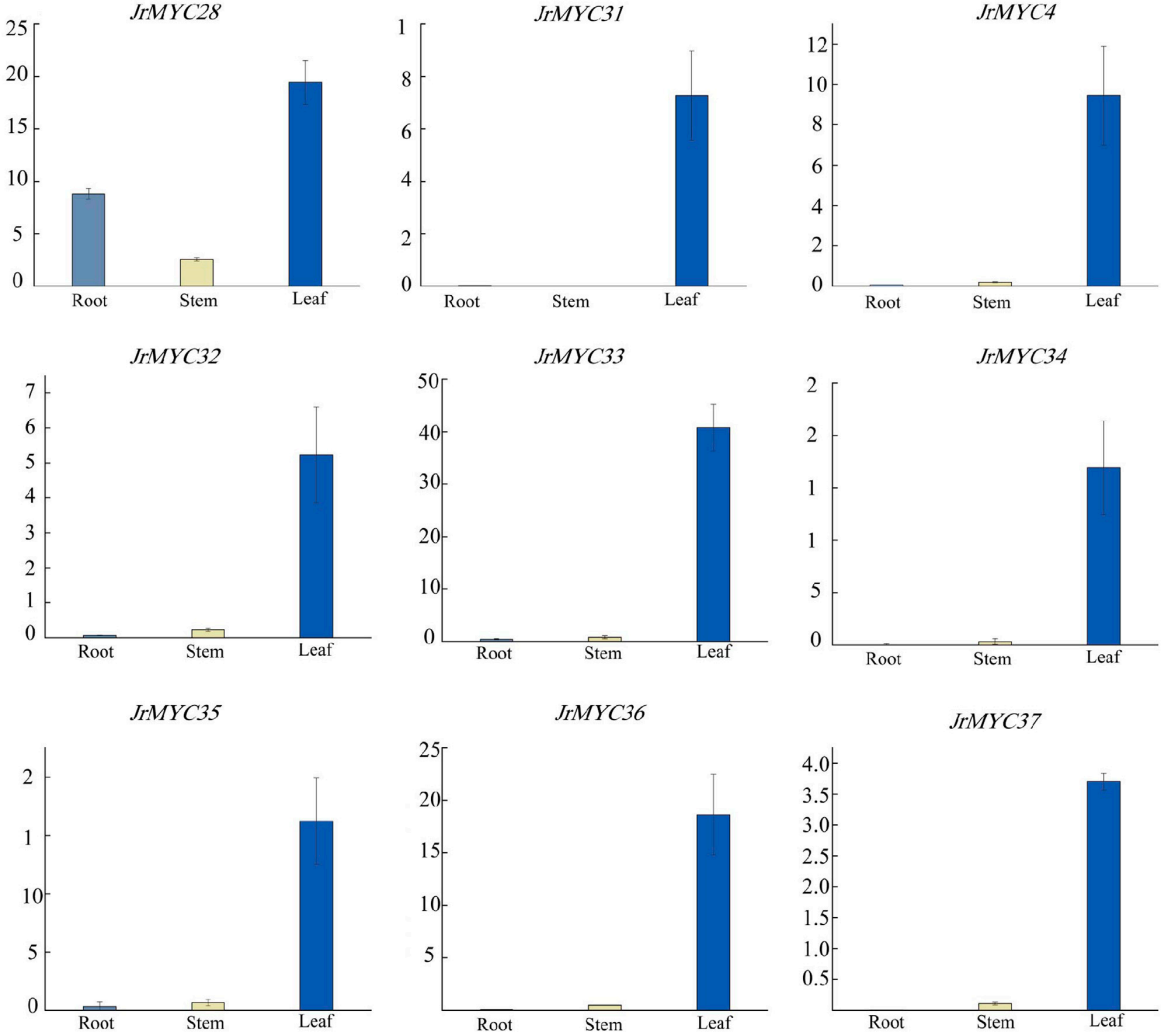
Expression levels of MYC transcription factor family members in different tissues of wild walnut in Xinjiang. Light blue represents roots, yellow represents stems, and blue represents leaves.

To investigate the expression patterns of the nine MYC transcription factor family members of Xinjiang wild walnut screened from transcriptome data in different tissues, the relative expression of each member in roots, stems and leaves was analysed (Figure 10; Figure 11). The results showed that each member of the MYC transcription factor family of Xinjiang wild walnut had the highest expression level in leaves, and the relative expression of *JrMYC28*, *JrMYC33*, *JrMYC34*, *JrMYC35*, and *JrMYC36* in leaves of Xinjiang wild walnut reached more than 15, which were upregulated genes, and the comprehensive analysis speculated that *JrMYC28*, *JrMYC33*, *JrMYC34*, *JrMYC35* and *JrMYC36* genes may play a crucial role in the regulation in response to low temperature stress.

## 4 Discussion

Phytohormones play a key role in adverse stress conditions, and jasmonic acid is an important phytohormone in plants. JA and its derivatives are important signaling molecules involved in the regulation of plant responses to adverse stresses such as low temperature, high salt and drought (Lyons et al., 2013; Fàbregas and Fernie, 2021; Raza et al., 2021). When plants are subjected to adverse conditions, the jasmonic acid signaling pathway activates related transcription factors to induce the expression of downstream functional genes, thereby increasing plant resistance to adverse conditions (Wang et al., 2020). MYC is an important component of the JAZ, COI1 and MYC2 complexes, which can mediate the generation of the jasmonic acid signaling pathway (Kazan and Manners, 2011). However, in recent years, studies on the gene characterization of the MYC transcription factor family in walnut and its response to adverse stress have not been reported. In this study, we identified each member of the MYC transcription factor family in walnut, predicted the physicochemical properties, phylogeny, conserved motifs, chromosomal localization, *cis*-acting elements, and protein interactions of the members, screened nine genes of the MYC transcription factor family in combination with the transcriptome data of the wild walnut in Xinjiang in response to low-temperature stress, and characterized the subcellular localization and expression patterns of these nine genes in different tissues and in response to low-temperature stress. The subcellular localization of these nine genes and their expression patterns in different tissues and in response to low-temperature stress were systematically analyzed to provide a theoretical foundation for further investigations of the functional mechanism of the MYC transcription factor family in walnut.

In this study, 30 MYC transcription factor family members were identified from the published walnut genome-wide database, and based on phylogenetic developmental analysis, the walnut MYC transcription factor family was categorized into six subgroups, with homology closer to 10 in poplar and 8 in Arabidopsis. In addition, phylogenetic development revealed that JrMYC22 and JrMYC23 are more closely related to PtrMYC2b. It has been shown that *PtrMYC2b* in poplar (Hu M. X. et al., 2023) is induced by jasmonic acid to respond to low temperatures to regulate the expression of downstream genes, thus improving plant resistance, and it is hypothesized that *JrMYC22* and *JrMYC23* may be induced by the jasmonic acid signaling pathway to activate downstream *cis*-acting element-specific binding to regulate the expression of functional genes in response to low-temperature stress (Figure 1). The 30 walnut MYC transcription factor family members in this study were located on 12 chromosomes, and the genes were unevenly distributed on the chromosomes, with significant differences in chromosome length and tandem duplications (Figure 2). Gene duplication is the main pathway for the amplification of gene family members, and tandem duplication is one of the important modes of gene duplication (Vision et al., 2000). Analysis revealed that there were nine genes in the walnut MYC transcription factor family with tandem duplication, namely, *JrMYC5, JrMYC7, JrMYC8, JrMYC9, JrMYC11, JrMYC14, JrMYC18, JrMYC24*, and *JrMYC25*, which accounted for 30% of the total number of genes in the gene family, and combined with the conserved structural domains, these genes had a high degree of similarity (Figure 4B). Thus, it was speculated that the walnut MYC transcription factor family may not have undergone large-scale gene expansion or relative conservation during evolution. Conserved motif analysis of each member of the walnut MYC transcription factor family revealed (Figure 4) that the conserved structural motifs of members located in the same subfamily were consistent, suggesting that the protein function of each member is stable. Most members of the walnut MYC transcription factor family have Motif7 and Motif8 and are highly conserved. It is hypothesized that most walnut MYC transcription factor family members may perform similar functions. The JrMYC12, JrMYC13, JrMYC15, JrMYC16, JrMYC17, and JrMYC18 proteins lacked Motif7 and Motif8, and it is hypothesized that these members may have different functions.

Promoter *cis*-acting elements are key for inducing functional gene expression. When plants are subjected to adverse stress, promoter *cis*-acting elements are involved in activating related transcription factors to regulate downstream gene expression in response to adverse environmental hazards (Nakashima et al., 2009; Wittkopp and Kalay, 2011). To clarify the functional characteristics of each member of the walnut MYC transcription factor family, we analyzed 30 members for promoter *cis*-acting element prediction and found that the promoter regions of the walnut MYC transcription factor family members contained *cis*-acting elements related to hormones (growth hormone, salicylic acid, gibberellin, jasmonic acid, abscisic acid), light signaling and low-temperature stress, which suggests that the walnut MYC family may be involved in the regulation of plant growth, development, and stress. Plant growth and development, adversity stress, biosynthesis and other biological processes. In addition, 18 genes encoded *cis*-acting elements involved in the response to low-temperature stress, among which jasmonic acid-related genes were the most widely distributed (12), and it was hypothesized that these genes might be induced by jasmonic acid in response to low-temperature stress, which is in line with the findings of studies on tomato (Miura et al., 2012), pomegranate (Sayyari et al., 2011), mango (González-Aguilar et al., 2000), and poplar (Hu et al., 2023). The predicted protein interactions of walnut MYC transcription factor family members revealed that walnut JrMYC25 and JrMYC29 had the strongest interactions and shared high homology with MYC2 and MYC3 in *Arabidopsis thaliana*, and MYC2 and MYC3 worked together to activate the relevant transcription factors of the jasmonic acid signaling pathway in response to adversity (Cheng et al., 2011). Similarly, the homologous protein of MYC2 in *Arabidopsis thaliana*, ICE1, could respond to low-temperature stress by inducing jasmonic acid in the transcriptional pathway. ICE1, a homologous protein of MYC2 in *Arabidopsis thaliana*, can induce the expression of downstream genes in response to low-temperature stress to enhance the cold resistance of plants (Lorenzo et al., 2004). In addition, it was stated that ICE1 may modulate cold-regulated (COR) genes in a CBF-dependent/independent way in plant species in response to low-temperature and cold stress (Heidari, 2019; Heidari et al., 2021). The homology between proteins can influence functional similarity, and it is hypothesized that JrMYC25 and JrMYC29 may have functions similar to those of MYC2 and MYC3 in *Arabidopsis thaliana* and may improve resistance in response to low-temperature stress, providing theoretical support for further studies on the mechanism underlying the response of walnut MYC transcription factor family members to cold stress. This study provides theoretical support for further exploration of the mechanism of MYC transcription factor family members in walnut plants in response to low-temperature stress.

In this study, nine MYC family members in response to low-temperature stress were screened based on the transcriptomic data of wild walnut plants in Xinjiang. By subcellular localization, it was found that the genes all undergo fluorescence signaling in the nucleus, among which *JrMYC4, JrMYC32, JrMYC33, JrMYC34, JrMYC35, JrMYC36, *and* JrMYC37* showed strong fluorescence responses, and combined with the expression patterns of these nine genes in response to low-temperature stress, *JrMYC4, JrMYC32, *and* JrMYC35* were expressed at relatively high levels at 8 h, which was consistent with the transcriptome data (Figure 8; Figure 9). It is hypothesized that *JrMYC28, JrMYC31, JrMYC33, JrMYC34, *and* JrMYC35* may play key roles in the response to low-temperature stress. The expression patterns of the nine genes in different tissues revealed that all of these genes were highly expressed in leaves, among which *JrMYC28, JrMYC33, JrMYC35*, and *JrMYC36* were highly expressed in wild walnuts in Xinjiang, and it was hypothesized that *JrMYC28, JrMYC33, JrMYC35*, and *JrMYC36* might play a key role in functional regulation (Figure 10; Figure 11). The results of this study provide theoretical support for further analysis of the functional mechanism of the MYC transcription factor family in walnut.

## 5 Conclusion

In summary, 30 MYC transcription factor family members were identified in walnuts in this study, and their physicochemical properties, phylogenetic relationships, chromosomal localization, sequence structures, promoter *cis*-acting elements and protein interactions were analyzed. The 30 identified MYC transcription factor family members were screened in combination with the transcriptome data of wild walnuts in Xinjiang in response to low-temperature stress, and the subcellular localization and expression patterns of these members were analyzed under low-temperature stress and in different tissues. The results showed that these members have the bHLH and bHLH_MYC_N structural domains of the MYC transcription factor family and are closely related to those of *Arabidopsis thaliana* and poplar. Among them, *JrMYC22* and *JrMYC23* have high homology with *PtrMYC2b*, which is induced by jasmonic acid in response to low-temperature stress to improve the cold tolerance of plants, and it is speculated that *JrMYC22* and *JrMYC23* may play key roles in low-temperature stress. Chromosomal localization revealed that walnut MYC members were unevenly distributed across 12 chromosomes, with possible tandem duplications of *JrMYC9, JrMYC11, JrMYC5, JrMYC14, JrMYC18, JrMYC24, JrMYC25, JrMYC7* and *JrMYC8*. Based on the sequence structure of walnut, it was found that most of the members may have functional redundancy and that a few members may have functionally different response mechanisms. The promoter *cis*-acting elements revealed that most of the walnut MYC genes were correlated with hormone *cis*-acting elements, among which the *cis*-acting elements related to jasmonic acid and low temperature were the most widely distributed, with 12 of them. Protein interactions predicted for walnut MYC members revealed that JrMYC25 and JrMYC29 had the greatest strength of interactions and high homology with MYC2 and MYC3 in *Arabidopsis thaliana*, and it was hypothesized that JrMYC25 and JrMYC29 might be able to regulate the jasmonic acid signaling pathway in response to low-temperature stress. In addition, nine MYC members were screened for subcellular localization using transcriptome data from Xinjiang wild walnut plants, and the results revealed that these members emitted strong fluorescent signals in the nucleus. According to the analysis of these members in response to low-temperature stress, the expression of *JrMYC28, JrMYC31, JrMYC33, JrMYC34,* and *JrMYC35* increased, suggesting that these three members might play important roles in the response to low-temperature stress. From the expression levels of the nine MYC members in different tissues, it was found that all of these members were highly expressed in leaves, among which *JrMYC28, JrMYC33*, *JrMYC35* and *JrMYC36* were significantly upregulated in wild walnut in Xinjiang, and it was speculated that these three members might play important roles in this process. The results of this study provide a theoretical basis for further research on the functional mechanisms of the MYC transcription factor family members in walnut.

## Data Availability

The datasets presented in this study can be found in online repositories. The names of the repository/repositories and accession number(s) can be found in the article/Supplementary Material.
